# Agreement Between Transthoracic Echocardiography and Digital Subtraction Angiography for the Detection of Right Atrial Thrombus in Patients Undergoing Maintenance Hemodialysis: A Retrospective Cohort Study

**DOI:** 10.3390/jcm15156084

**Published:** 2026-08-05

**Authors:** Fen Yu, Xiaomei Huang, Jingjing Liu, Wei Xiao, Qiao Huang, Jianxin Liu

**Affiliations:** 1 Department of Ultrasound, The Central Hospital of Wuhan, Tongji Medical College, Huazhong University of Science and Technology, Wuhan 430014, China; yufen@zxhospital.com (F.Y.); liujingjing1@zxhospital.com (J.L.); 2 Key Laboratory for Molecular Diagnosis of Hubei Province, The Central Hospital of Wuhan, Tongji Medical College, Huazhong University of Science and Technology, Wuhan 430014, China; huangxiaomei@zxhospital.com (X.H.); xiaowei@zxhospital.com (W.X.); huangqiao@zxhospital.com (Q.H.); 3 Department of Nephrology, The Central Hospital of Wuhan, Tongji Medical College, Huazhong University of Science and Technology, Wuhan 430014, China

**Keywords:** maintenance hemodialysis, right atrial thrombus, transthoracic echocardiography, digital subtraction angiography, risk factors

## Abstract

**Background/Objectives**: Patients on maintenance hemodialysis (MHD) have an increased risk of catheter-related right atrial thrombosis (CRAT) due to long-term central venous catheterization. Transthoracic echocardiography (TTE) and digital subtraction angiography (DSA) are commonly used to evaluate right atrial thrombi, yet their diagnostic consistency in this patient population remains unclear. This study aimed to compare TTE and DSA diagnostic agreement for CRAT and explore its independent risk factors. **Methods**: We retrospectively enrolled 254 MHD patients who underwent paired same-day TTE and DSA from November 2021 to May 2025. Cohen’s kappa, intra-class correlation coefficient (ICC), and Bland–Altman analysis were used to evaluate diagnostic consistency. Univariable and multivariable logistic regression screened independent CRAT risk factors. **Results**: The thrombus detection rates of TTE and DSA were 17.3% and 14.6%, respectively, with moderate diagnostic consistency (*κ* = 0.487, *p* < 0.001). The ICC values were 0.871 for thrombus length and 0.824 for thrombus width between TTE and DSA, indicating excellent consistency in thrombus size measurement between the imaging modalities. Catheter tip located in the mid-right atrium, decreased central venous catheter flow, prolonged catheterization, and male sex were independent risk factors for CRAT. The predictive model exhibited good discrimination (area under the curve = 0.788) and acceptable calibration (Hosmer–Lemeshow, *p* = 0.429). The optimal cutoff derived from the Youden index was 0.191, with a corresponding sensitivity of 81.0% and specificity of 67.3%. **Conclusions**: TTE is recommended as the first-line CRAT screening tool for MHD patients. Combined TTE and DSA use improves diagnostic yield. Regular targeted surveillance for high-risk individuals reduces CRAT incidence.

## 1. Introduction

The 2019 KDOQI Vascular Access Guideline advocated a “patient first” philosophy and recommended autogenous arteriovenous fistula (AVF) as the preferred long-term vascular access, while restricting the use of central venous catheters (CVCs) for chronic hemodialysis. Non-tunneled CVCs have been approved only for short-term use (<2 weeks). Tunneled CVCs are reserved for patients with repeated AVF failure, no alternative vascular access, or limited life expectancy [1]. Despite these rigorous recommendations, a considerable proportion of patients undergoing maintenance hemodialysis (MHD) remain CVC-dependent. In China, more than 50% of patients receiving MHD undergo CVC placement within the first 60 days of dialysis initiation, and approximately 12% rely on CVCs for permanent vascular access [2]. Guidelines suggest positioning the catheter tip in the mid-right atrium to ensure adequate blood flow and mitigate catheter-related vascular injury [1]. However, this anatomical location inevitably increases the risk of right atrial thrombosis and cardiac arrhythmias.

Catheter-related right atrial thrombosis (CRAT) has been reported in 2–29% of patients with indwelling CVCs. However, its true incidence is substantially underestimated as a large number of affected individuals present with no obvious symptoms. CRAT may lead to severe complications including pulmonary embolism, septic embolism, cardiac dysfunction, and systemic embolism. A systematic review of 144 CRAT cases reported an overall CRAT-related mortality rate of 18.1% [3].

CRAT carries substantial life-threatening complications, yet standardized diagnostic guidelines specifically targeting this entity remain absent, highlighting an urgent demand for evidence-driven diagnostic workflows. Transthoracic echocardiography (TTE) is the first-line screening modality because of its non-invasiveness and ability to visualize thrombus morphology and mobility in real-time [4]. Transesophageal echocardiography (TEE) is a valuable supplementary tool; however, its semi-invasive nature precludes its routine clinical use [5]. A recent systematic review of catheter-related thrombosis notes that cardiac computed tomography (CT) and magnetic resonance imaging (MRI) serve as second-line tools for patients with inconclusive TTE findings [6]. As the diagnostic gold standard for central venous lesions, digital subtraction angiography (DSA) is routinely applied for diagnosis and interventional management in patients receiving MHD; it also facilitates the assessment of right atrial thrombi [7].

However, most existing studies on CRAT have focused on general hospitalized cohorts, whereas systematic head-to-head comparisons between TTE and DSA remain limited, particularly in populations receiving MHD. The qualitative and quantitative diagnostic agreement between imaging modalities, their unique advantages in identifying different thrombus phenotypes, and independent risk factors for CRAT among patients receiving MHD are yet to be fully elucidated. Addressing these unmet research questions is essential for establishing evidence-based CRAT screening protocols and optimizing routine clinical screening workflows for hemodialysis patients. Therefore, this single-center retrospective study was designed with three main objectives: (i) to compare the diagnostic agreement between TTE and DSA for right atrial thrombus in patients receiving MHD; (ii) to identify independent risk factors for CRAT development; and (iii) to assess the discriminative performance of a logistic regression model constructed using the above risk factors.

## 2. Materials and Methods

### 2.1. Study Population

This retrospective study consecutively enrolled patients undergoing MHD in the nephrology department of our hospital. All patients underwent central venous DSA for vascular access-related issues between November 2021 and May 2025.

Inclusion criteria:

1. Patients receiving regular MHD with at least one documented CVC placement;
2. Patients who underwent central venous DSA for clinically indicated vascular access assessment;
3. Patients who completed TTE on the same day as DSA.

Exclusion criteria:

Patients with incomplete ultrasound archives or images that could not be interpreted due to non-standard storage. Ultimately, 254 patients with complete baseline clinical and imaging data were included.

**Figure 1:**
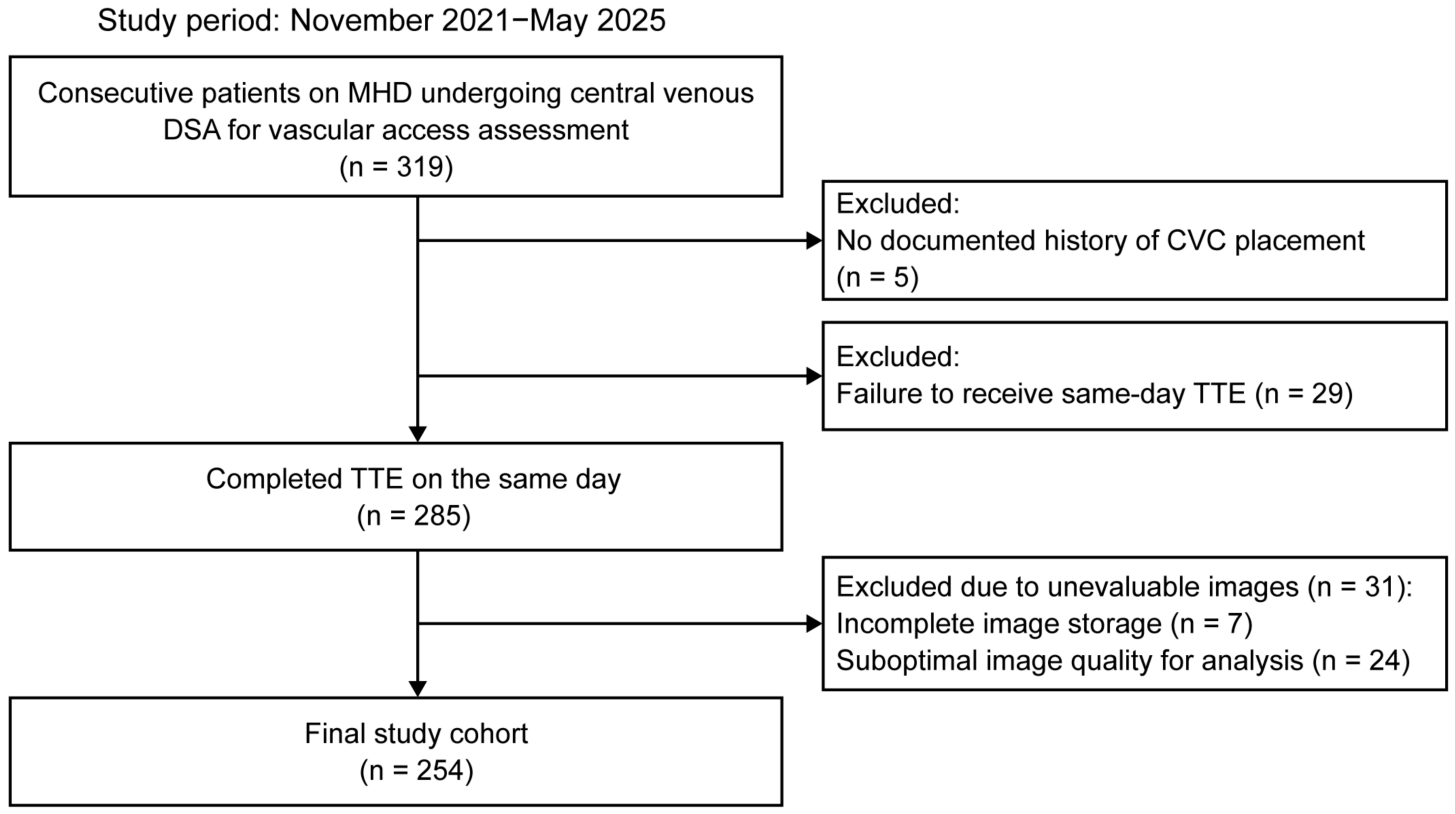
Flowchart of participant selection. Study enrollment spanned from November 2021 to May 2025. A total of 319 consecutive patients receiving MHD underwent central venous DSA for vascular access assessment; five patients without history of CVC placement and 29 patients lacking same-day TTE were initially excluded, leaving 285 patients with paired imaging. An additional 31 patients were excluded owing to unevaluable images: seven cases had incomplete image storage, and 24 datasets presented suboptimal image quality for analysis. These 24 datasets were excluded due to pulmonary gas shadowing, inadequate patient positional cooperation, and weakened ultrasonic penetration resulting from chest wall edema and thickened subcutaneous tissue, all of which interfered with precise identification and quantitative measurement of right atrial thrombi. The final cohort comprised 254 patients. Abbreviations: CVC, central venous catheter; DSA, digital subtraction angiography; MHD, maintenance hemodialysis; TTE, transthoracic echocardiography.

A total of 319 consecutive patients on MHD were initially screened. After stepwise exclusion of individuals who did not meet the above criteria, 254 patients with complete baseline clinical and imaging data were finally enrolled in the study. The complete participant screening and exclusion process is summarized in Figure 1.

This study was approved by our Institutional Ethics Review Committee (approval no. WHZXKYL2025-094) and was conducted in accordance with the principles of the Declaration of Helsinki. The requirement for written informed consent was waived because all data were retrieved from anonymized archived medical records.

### 2.2. Examination Methods

#### 2.2.1. TTE Examination

All TTE examinations were performed using a Philips EPIQ 7C (Philips Medical Systems, Suzhou Co., Ltd.; Suzhou, China) with an S5-1 transducer (frequency range 1–5 MHz). The patients were examined in the supine or left lateral decubitus position. Multiple standard views were sequentially acquired, including the parasternal long-axis view of the ascending aorta and parasternal right ventricular inflow, apical four-chamber, apical five-chamber, subcostal superior-inferior vena cava, and subcostal four-chamber views. The right atrium, indwelling CVC, and superior vena cava orifice were systematically evaluated.

The main observational parameters were as follows:

(1) Presence of abnormal echo lesions within the right atrium or catheter surface.
(2) For detected lesions, the maximum length, maximum width, and mobility pattern (limited mobility or free-floating) were recorded.
(3) Anatomical location of the CVC tip.

#### 2.2.2. DSA Examination

DSA was performed using a CGO-2100 digital angiography system (Wandong Medical Equipment, Beijing, China). All patients were placed in the supine position. A 20-gauge venous puncture needle was inserted bilaterally into the median cubital veins for contrast administration. Non-ionic iodinated contrast medium (iohexol, Omnipaque, GE Healthcare Pharmaceutical (Shanghai) Co., Ltd., Shanghai, China) was injected via the peripheral veins of the upper extremities. For patients with cervical CVC, the contrast medium was delivered simultaneously through the catheter.

Images were acquired during end-inspiratory breath-holding. The contrast medium was injected using a high-pressure injector at a rate of 5 mL/s. The single bolus dose was 15–20 mL, and the total contrast volume for each patient ranged from 40 to 60 mL. Conventional anteroposterior sequential images were also acquired.

The main observational parameters included the presence, size, and morphology of filling defects in the right atrium and anatomical position of the CVC tip.

#### 2.2.3. Image Interpretation and Measurement

TTE images were independently analyzed by two echocardiographers with 19 and 15 years of clinical experience, respectively. DSA images were independently assessed by two interventional nephrologists (with 15 and 9 years of experience, respectively) using Anythink EP 4.2 image analysis software (Keruilai Fu Technology, Beijing, China). All interpreters were blinded to the results of the other imaging modality.

Intraclass correlation coefficients (ICCs) and Bland–Altman analyses were used to separately evaluate inter-observer agreement on thrombus length and width for TTE and DSA. One reader for each modality repeated the measurements after a 4-week interval to assess intra-observer reproducibility. The average of the two readers’ measurements was used for the final analysis, and inconsistent readings were resolved by consensus. For categorical endpoints, including CRAT presence and thrombus mobility, only cross-modality Cohen’s kappa was calculated, given objective binary diagnostic standards, without additional reader-to-reader reliability testing.

Notably, DSA identifies thrombotic lesions indirectly as contrast filling defects on subtracted angiography. Thin mural thrombi often fail to produce sufficiently discernible filling defects, and free-floating thrombi shift with cardiac motion, rendering them difficult to capture on DSA images. In contrast, TTE directly visualizes right atrial masses in real time, clearly depicting thrombus morphology and mobility. Therefore, to avoid underestimating the true prevalence of CRAT due to the limitations of a single imaging technique, we adopted a composite diagnostic endpoint: a patient was defined as CRAT-positive if thrombus was identified on either TTE or DSA.

### 2.3. Clinical Data Collection

Relevant data were extracted from the hospital’s electronic medical record and image archiving systems, including demographic characteristics (age, sex, height, body weight, and blood pressure), dialysis duration, and CVC-related information (total catheterization time, number of catheter insertions, insertion site, catheter tip position, and catheter type). The primary clinical presentation of each patient was also recorded.

### 2.4. Definitions

TTE+ refers to a right atrial thrombus detected on TTE. DSA+ refers to a right atrial thrombus detected on DSA. A thrombus with limited mobility comprised a solid mass without flocculent echoes showing no obvious movement or only slight swinging against the right atrial wall or CVCs. A free-floating thrombus comprised a mass lesion with surface flocculent echoes and a wide range of mobility within the right atrium or CVCs.

### 2.5. Statistical Analysis

All statistical analyses were performed using SPSS 30.0 (IBM Corp., Armonk, NY, USA). Statistical significance was set at two-tailed *p* < 0.05. Continuous data were tested for normality using the Shapiro–Wilk test; normally distributed data are presented as mean ± SD and were compared using an independent samples t-test, while non-normal data are reported as median (interquartile range; IQR) and were compared using the Mann–Whitney U test or Kruskal–Wallis H test with Bonferroni post-hoc correction. Categorical data are presented as *n* (%) and were compared using the χ^2^ test or Fisher’s exact test as appropriate. The cross-modality diagnostic agreement between TTE and DSA was assessed using Cohen’s kappa (95% CI) for binary thrombus detection outcomes, whereas ICCs paired with Bland–Altman analyses were used for quantitative thrombus dimensional measurements. Separate ICC calculations were also conducted to evaluate the inter-observer and intra-observer reliability of thrombus length and width for both TTE and DSA images. Binary logistic regression (Enter method) was used to identify independent risk factors for CRAT, and a positive outcome was defined as a thrombus detected using either imaging modality.

This composite endpoint was adopted instead of DSA alone to minimize missed thrombotic events and ensure sufficient positive cases for robust logistic regression analysis. All predictors with *p* < 0.05 in univariate comparisons formed the candidate variable pool. We selected four core variables (catheter tip position, sex, clinical presentation, and total catheterization time) for multivariate modelling. During variable selection, we prioritized indicators with direct pathogenic relevance to CRAT; among these, variables with the strongest between-group discriminatory performance were retained. Given the limited number of thrombotic endpoints (58 events among 254 participants), several statistically significant predictors were excluded to optimize the EPV value and reduce the risk of model overfitting.

## 3. Results

### 3.1. Patient Baseline Characteristics

Of the 254 patients undergoing MHD, 109 (42.9%) were male. The median age was 66.0 years (IQR 54.0–74.0), and the median dialysis vintage was 33.5 months (IQR 9.8–74.3). All patients had prior cervical CVC placement (range 1–6 insertions), 119 (46.9%) had ≥2 insertions, and 10 (3.9%) had bilateral placement. At enrollment, 218 patients (85.8%) had an indwelling cervical CVC, mostly via the right internal jugular vein (204, 80.3%). Long-term catheters were predominantly used (198, 90.8%; step-tip 103, symmetric-tip, 83). Among the patients with an indwelling catheter, 140 (64.2%) had the tip in the right atrium (135 long-term, five temporary). The primary reasons for examination were reduced CVC flow (100, 39.4%), other nonobstructive causes (128, 50.4%), and edema/varicose veins (26, 10.2%) (Table 1).

### 3.2. Thrombus Detection Rates and Agreement

TTE and DSA detected thrombi in 44 (17.3%) and 37 patients (14.6%), respectively (Table 2). Cohen’s kappa was 0.487 (95% CI: 0.321–0.653, *p* < 0.001), indicating moderate agreement (Landis and Koch). McNemar’s test yielded *p* = 0.311, indicating no significant difference in the detection rates or systematic bias.

### 3.3. Agreement in Thrombus Size Measurement

**Figure 2:**
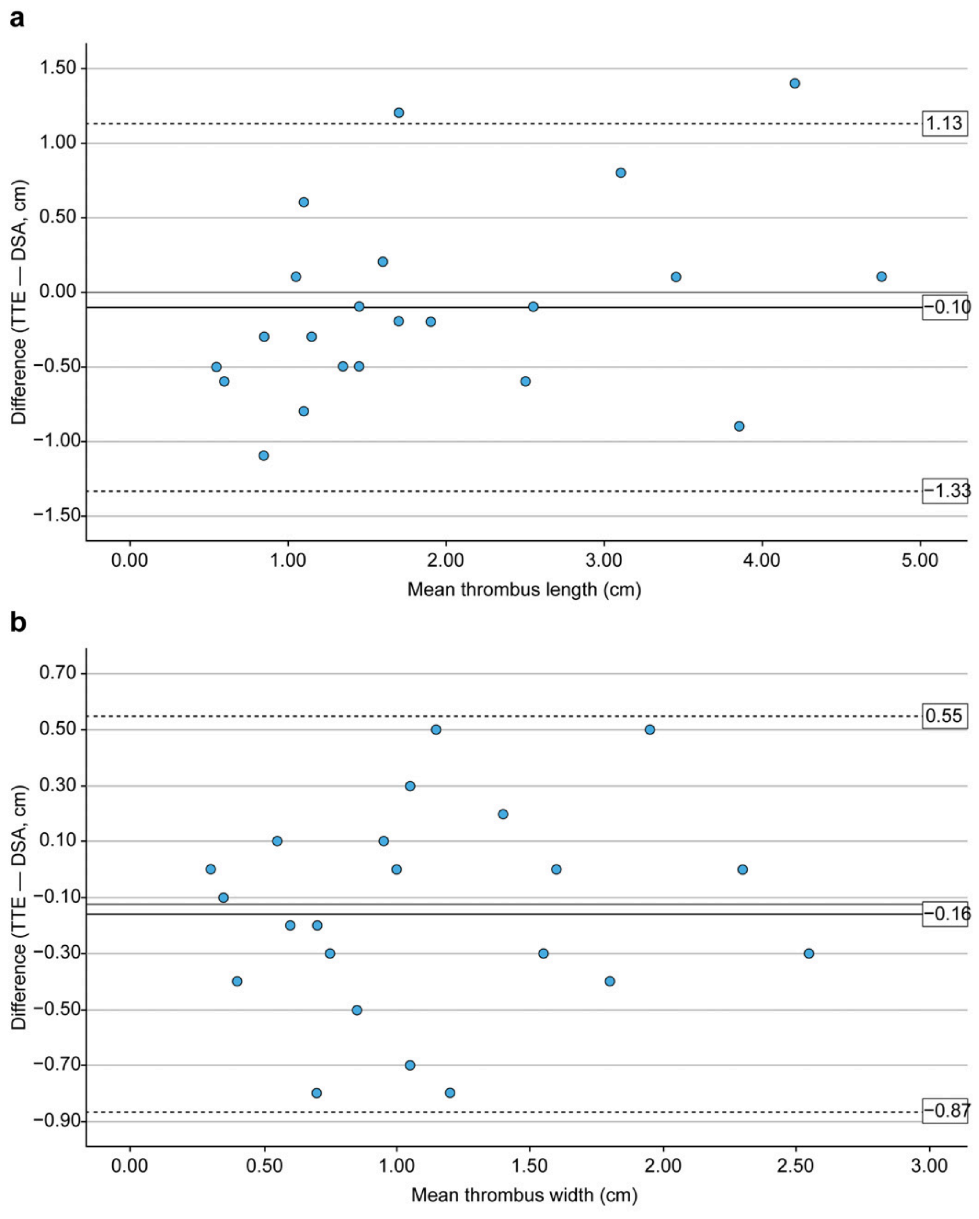
Bland–Altman plots for consistency of thrombus length (**a**) and width (**b**) measured using TTE versus DSA. (**a**) Thrombus length. The mean difference between TTE and DSA was −0.10 cm, with 95% limits of agreement ranging from −1.33 to 1.13 cm; 91.3% of data points fell within the 95% limits of agreement. (**b**) Thrombus width. The mean difference was −0.16 cm, with 95% limits of agreement from −0.87 to 0.55 cm; all data points were within the 95% limits of agreement. Solid line = mean difference; dashed lines = 95% limits of agreement. DSA, digital subtraction angiography; TTE, transthoracic echocardiography.

Bland–Altman analysis (Figure 2) was used to assess quantitative agreement. For the 23 patients with measurable thrombi on both modalities, the ICC for length was 0.871 (95% CI, 0.723–0.943; *p* < 0.001). The mean bias was −0.10 cm (95% limits of agreement −1.33 to 1.13 cm); 21/23 (91.3%) differences fell within these limits. For width, ICC was 0.824 (95% CI, 0.612–0.923; *p* < 0.001), and the mean bias was −0.16 cm (95% limits of agreement −0.87 to 0.55 cm), with all differences within the limits.

### 3.4. Thrombus Length and Width Across Detection Patterns

**Figure 3:**
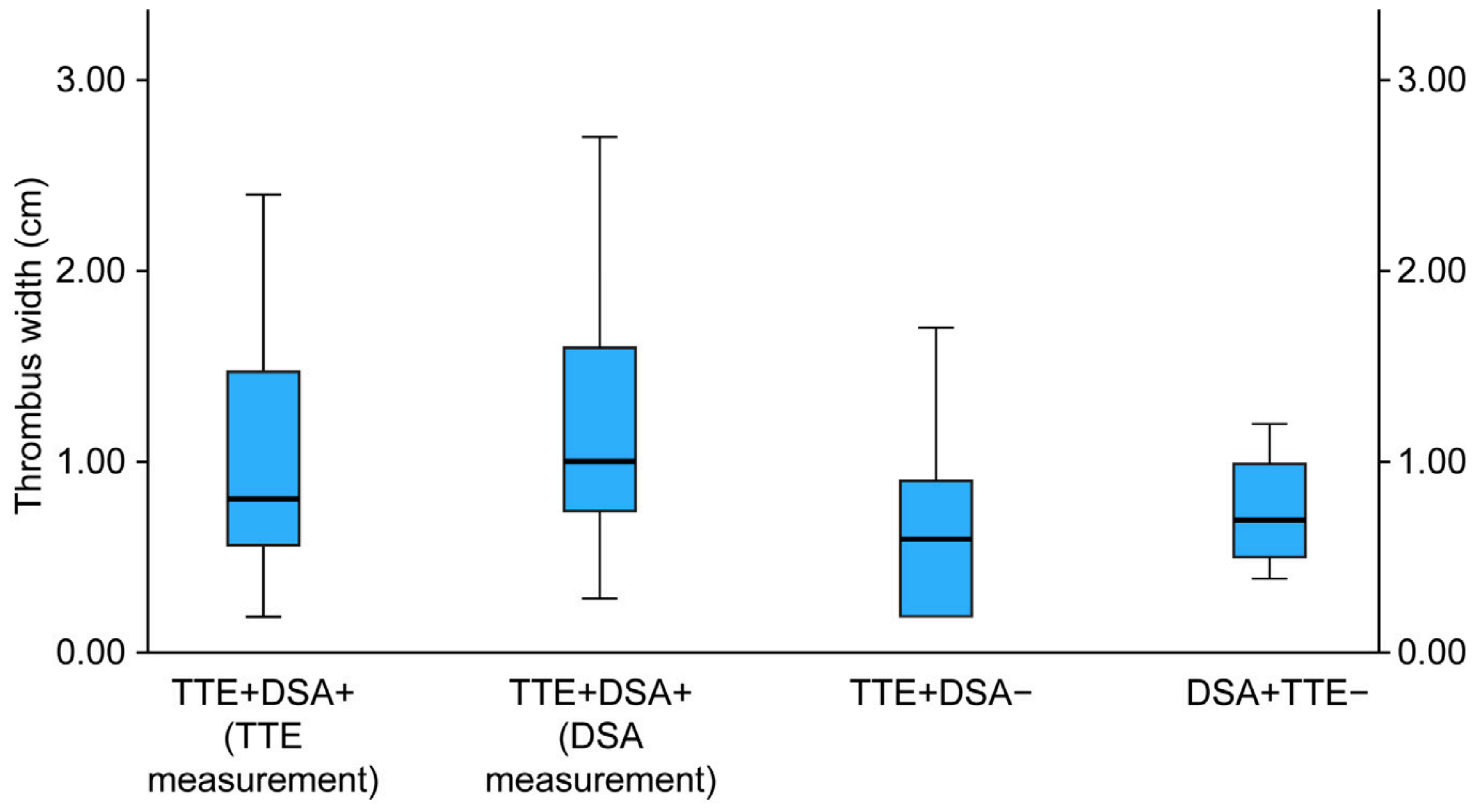
Box-and-whisker plot illustrating the distribution of thrombus width (cm) in four imaging detection subgroups. Note: The Kruskal–Wallis H test was used for intergroup comparisons of thrombus width; post-hoc Bonferroni correction revealed that thrombus width in the TTE+/DSA− group was significantly lower than that measured by DSA in the TTE+/DSA+ subgroup (adjusted *p* = 0.017). DSA, digital subtraction angiography; TTE, transthoracic echocardiography.

Four subgroups were compared: TTE+/DSA−, DSA+/TTE−, TTE+/DSA+ (TTE measurement), and TTE+/DSA+ (DSA measurement). Length did not significantly differ (H = 5.307, df = 3, *p* = 0.151) but width did (H = 9.959, df = 3, *p* = 0.019). Bonferroni-corrected pairwise comparisons showed that width in the TTE+/DSA− group was significantly smaller than DSA-measured width in the TTE+/DSA+ group (adjusted *p* = 0.017); no other pairwise differences were significant (Figure 3).

### 3.5. Influence of TTE-Assessed Thrombus Mobility on DSA Detection

Of the 44 TTE-positive thrombi, 36 had limited mobility and eight were free-floating. Free-floating thrombi were significantly longer [2.60 cm (1.33–3.40) vs. 1.15 cm (0.30–1.78), *p* = 0.030] but not wider [0.90 cm (0.65–1.20) vs. 0.60 cm (0.23–1.18), *p* = 0.286]. The DSA detection rates were 55.6% (20/36) and 37.5% (3/8) for limited mobility and free-floating thrombi, respectively. No statistical tests were performed for the free-floating subgroup because of the small sample size, and the trend is reported descriptively.

### 3.6. Risk Factors for Right Atrial Thrombus

Using combined detection (TTE or DSA), 58 patients (22.8%) tested positive and 196 (77.2%) tested negative. In the univariate analysis, female patients had a higher positive rate than male patients (29.7% vs. 13.8%; χ^2^ = 8.921, *p* = 0.003). No other demographic differences were observed between the groups. Catheter-related factors (number of insertions, location, type, tip design, and tip position) were all significantly associated (*p* < 0.05). Tip position showed the strongest difference (χ^2^ = 30.573, *p* < 0.001); patients with the tip in the mid-RA had the highest rate (45.2%). Clinical presentation was also correlated (χ^2^ = 14.181, *p* < 0.001); reduced CVC flow yielded a 35.0% positive rate. Dialysis duration, total catheterization time, and current indwelling time were all longer in the positive group (all *p* < 0.05).

### 3.7. Independent Risk Factors and Model Performance

The logistic regression analysis included four core variables (tip position, sex, clinical presentation, and total catheterization time). The model was significant (omnibus χ^2^ = 46.840, df = 6, *p* < 0.001) with 79.1% overall accuracy. Independent risk factors included tip in mid-RA (odds ratio (OR) = 7.052, 95% confidence interval (CI) 3.012–16.513, *p* < 0.001), tip in upper/lower right atrium (OR = 2.564, 95% CI 1.097–5.993, *p* = 0.030), reduced CVC flow (OR = 2.352, 95% CI 1.164–4.755, *p* = 0.017), longer total catheterization time (OR = 1.011, 95% CI 1.001–1.021, *p* = 0.024), and male sex (OR = 2.052, 95% CI 1.007–4.180, *p* = 0.047). Tip in mid-RA was the strongest predictor.

**Figure 4:**
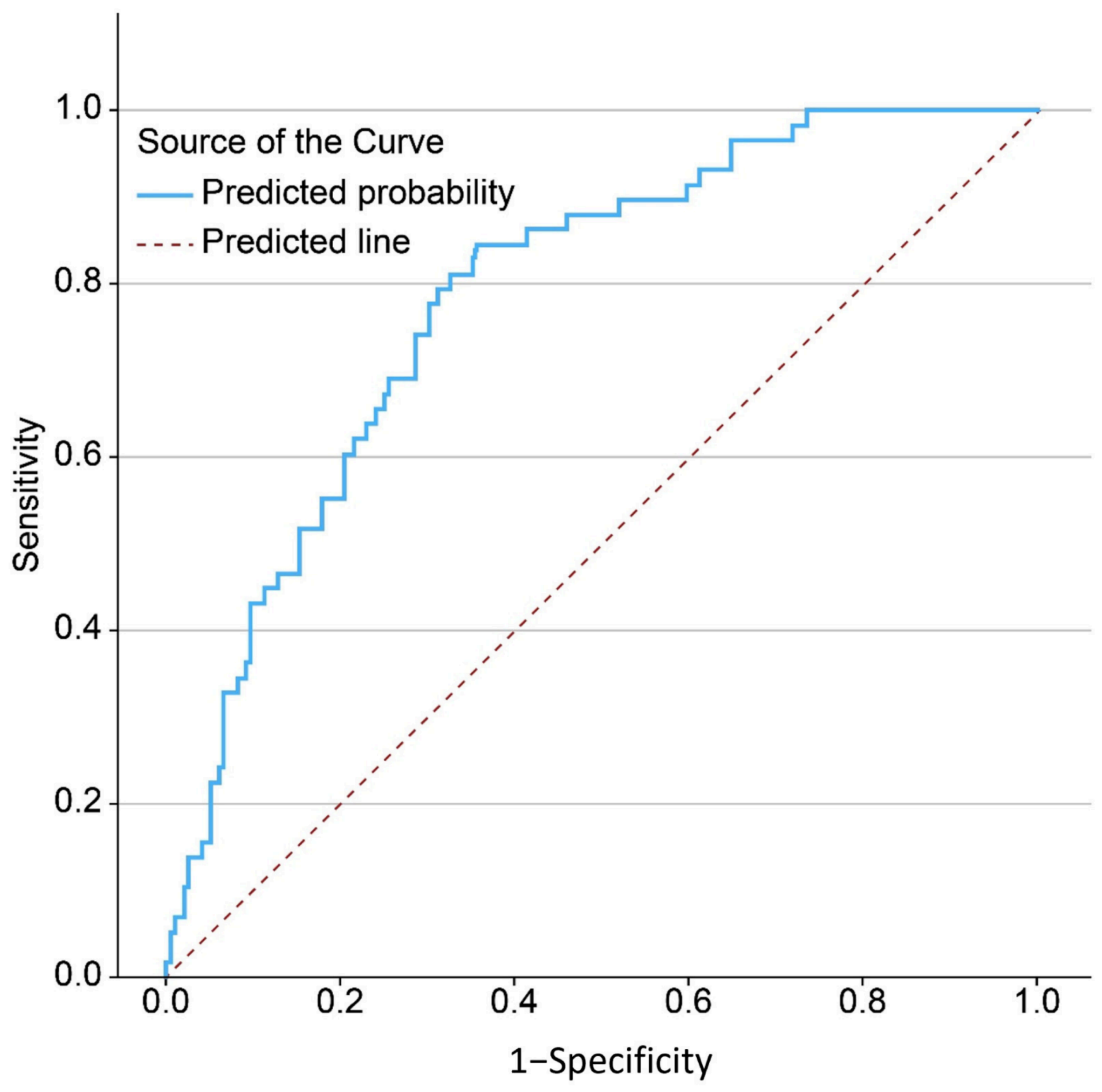
Receiver operating characteristic curve of the logistic prediction model. The area under the curve was 0.788 (95% confidence interval 0.726–0.849, *p* < 0.001). The optimal cutoff value derived from the Youden index was 0.191, with corresponding sensitivity of 81.0% and specificity of 67.3%.

The model showed good discrimination (area under the curve = 0.788, 95% CI 0.726–0.849, *p* < 0.001; Figure 4) and acceptable calibration (Hosmer–Lemeshow χ^2^ = 8.045, df = 8, *p* = 0.429). At a default cutoff of 0.5, the sensitivity and specificity were 32.8% and 92.9%, respectively. The optimal cutoff (Youden index) was 0.191, yielding a sensitivity of 81.0% and specificity of 67.3%.

### 3.8. Intra- and Inter-Observer Measurement Reliability

Intraclass correlation analysis (ICCs, single measures, two-way mixed absolute-agreement model) was used to assess the inter-observer agreement and intra-observer reproducibility of thrombus length and width measured on TTE and DSA.

For TTE (*n* = 44), the inter-observer ICC was 0.906 (95% CI, 0.834–0.948; *p* < 0.001) for length and 0.854 (95% CI, 0.748–0.918; *p* < 0.001) for width, and the intra-observer ICCs were 0.910 and 0.921 for length and width, respectively (all *p* < 0.001).

For DSA (n = 37 evaluable lesions), the inter-observer ICC was 0.817 (95% CI, 0.673–0.902; *p* < 0.001) for length and 0.876 (95% CI, 0.772–0.934; *p* < 0.001) for width, while the intra-observer ICCs for length and width were 0.948 and 0.956 (all *p* < 0.001), respectively.

All ICCs were >0.75, indicating acceptable to excellent measurement consistency across both imaging modalities.

## 4. Discussion

This retrospective study analyzed the clinical and imaging data of 254 patients receiving MHD. We characterized the ultrasonographic features of right atrial thrombi, compared the diagnostic agreement between TTE and DSA, and identified independent risk factors, providing evidence to optimize screening strategies.

TTE detected thrombi in 17.3% and DSA in 14.6% of patients (*p* = 0.311). Cohen’s kappa showed moderate agreement (κ = 0.487, *p* < 0.001), indicating that the two methods are not interchangeable. The combined detection rate was 22.8%, which is consistent with those reported by previous studies [8]. Both modalities showed excellent quantitative consistency in terms of thrombus size (ICC > 0.8). Bland–Altman analysis confirmed that nearly all measurement deviations fell within the 95% limits of agreement, verifying complementarity.

The discrepancy in detection rates stems from their inherent imaging properties. DSA is limited in visualizing thin mural and freely mobile intracardiac masses, whereas these lesions can be continuously tracked on dynamic TTE imaging. In our cohort, thrombus width in the TTE+/DSA− group was significantly smaller than that in the double-positive group (*p* = 0.017), suggesting that TTE has a higher sensitivity for thin thrombi. In standard TTE views (parasternal long-axis, right ventricular inflow, and subcostal four-chamber), the ultrasound beam is nearly perpendicular to the catheter and posterior right atrial wall, enabling clear visualization of mural thickening. Although DSA is the gold standard for central venous lesions [9,10], it has limitations. Thin mural thrombi often lack typical filling defects. Anatomical overlap, catheter signal interference, and rapid blood flow-diluting contrast agents collectively reduce image quality and cause missed diagnoses.

The 1989 European classification defined three right heart thrombus types: A (highly mobile), B (immobile mural), and C (intermediate). Type A has a higher pulmonary embolism risk than B [11]. Our thrombi were types B and C. Type C (free-floating) thrombi, despite their greater length, were less often detected with DSA. TTE enables real-time dynamic observation, conferring advantages to free-floating lesions, whereas DSA cannot capture stable defects. Therefore, TTE is more suitable for follow-up purposes.

Univariate analysis showed that female sex, number of insertions, catheter location, catheter type, tip design, tip position, clinical presentation, dialysis vintage, and total and current indwelling times were all significantly associated with thrombus formation (all *p* < 0.05). Logistic regression analysis confirmed that tip position, reduced flow, and prolonged catheterization were independent risk factors.

Mid-right atrial tip placement was the strongest risk factor (OR = 7.052, *p* < 0.001), whereas upper/lower placement conferred a lower risk (OR = 2.564, *p* = 0.03). Anatomically, the mid-right atrium has the largest transverse diameter; the catheter swings with the cardiac/respiratory cycles, causing endothelial injury. Hemodynamically, catheter placement disrupts normal vortical flow [12], and the mid-right atrium is the main buffer zone for caval return. Uremia-related volume overload, cardiac dilatation, and hypercoagulability synergistically elevate the risk, fulfilling Virchow’s triad.

Prolonged catheterization exacerbates this risk. Most CRATs are type B and asymptomatic, with reduced CVC flow being the earliest sign. In our cohort, patients with reduced CVC flow had higher thrombus detection rates (35.0%; OR, 2.352; *p* = 0.017). Clinically, a reduced CVC flow in patients with right atrial tip should prompt immediate imaging.

Notably, sex displayed divergent correlations with CRAT before and after multivariate adjustment, which can be categorized into two independent mechanisms: catheter access-related clinical dependency and inherent biological thrombotic risk.

First, univariate analysis revealed a higher crude CRAT detection rate among female MHD patients. As reported in a previous systematic review, women are more prone to autologous arteriovenous fistula maturation failure, leading to longer and more frequent reliance on central venous catheters [13]. This unequal catheter exposure, arising from routine vascular access allocation patterns, largely accounts for the higher crude CRAT prevalence observed among female patients.

Second, after fully correcting for catheter placement, dwell time and other clinical confounders, male sex remained an independent risk factor for CRAT (OR = 2.052, 95% CI 1.007–4.180, *p* = 0.047). This adjusted result demonstrates that males carry greater biological thrombotic risk when exposed to identical catheter-induced endothelial injury. Uremic men tend to have disordered metabolism, poor adherence to anticoagulation regimens, and more prothrombotic lifestyle habits [14,15], all of which collectively increase their risk of intracardiac thrombus formation under equivalent mechanical vascular stimulation.

### 4.1. Clinical Implications

TTE should be the first-line screening for suspected thrombi, given its superiority for thin mural and free-floating lesions. DSA should be reserved for patients requiring central intervention or those with high suspicion despite negative TTE. High-risk patients (mid-right atrial tip) warrant regular TTE surveillance (every 3–6 months). Repositioning the tip away from the mid-right atrium, if feasible, may reduce the risk. Patients with prolonged catheterization should undergo timely conversion to AVF. Male patients require stricter metabolic control and adherence to anticoagulation therapy. Reduced CVC flow should trigger immediate TTE rather than empirical manipulation. Using the optimal cut-off of 0.191, clinicians can identify high-risk individuals (predicted probability ≥ 0.191) for targeted screening.

### 4.2. Limitations

This was a single-center, retrospective study with a limited sample size, and all patients underwent DSA for clinical reasons, which may limit the generalizability of our findings. Moreover, only TTE and DSA were compared, and other modalities (TEE, CT, and MRI) were not included. Therefore, a larger, multicenter, prospective validation with long-term follow-up is required. The small sample of free-floating thrombi precluded statistical comparison. As such, the results are descriptive and require verification.

## 5. Conclusions

This study compared the diagnostic performance of TTE and DSA for CRAT in patients on MHD. The two modalities showed moderate agreement for thrombus detection (κ = 0.487) but excellent consistency in size measurements (ICC > 0.80), indicating that they are complementary rather than interchangeable. DSA serves as the standard for evaluating venous anatomical abnormalities, whereas TTE affords dynamic advantages for detecting mobile intracardiac thrombi; combined assessment can lower CRAT underdiagnosis. TTE was more sensitive to thin mural and free-floating mobile thrombi, whereas DSA tended to miss longer floating lesions. Multivariable analysis identified mid-right-atrial tip (OR = 7.052), reduced CVC flow, prolonged catheterization, and male sex as independent risk factors. The prediction model achieved good discrimination (AUC = 0.788) and acceptable calibration (Hosmer–Lemeshow P = 0.429), with an optimal cutoff of 0.191. We recommend TTE as the first-line screening tool, with DSA reserved for interventional guidance or cases of high suspicion despite negative TTE. Regular surveillance is advised for patients with mid-right atrial catheter tips, prolonged catheter indwelling, or reduced CVC flow to facilitate early risk stratification and reduce CRAT risk.

## Figures and Tables

**Table 1:** Clinical Characteristics of the Study Population (*n* = 254).

Variables	Values
Sex (male), *n* (%)	109 (42.9%)
Age (median, years)	66.0 (54.0, 74.0)
Height (median, cm)	160.0 (156.0, 169.0)
BMI (median, kg/m^2^)	22.0 (19.5, 25.0)
Systolic pressure (median, mmHg)	141.0 (125.0, 155.0)
Diastolic pressure (mean, mmHg)	81.3 ± 13.6
Hemodialysis duration (median, months)	33.5 (9.8, 74.3)
Total duration of CVC catheterization (median, months)	21.5 (6.8, 42.3)
Indwelling time of current in-situ CVC (median, months)	11.0 (2.0, 27.0)
Number of CVC placements	1.0 (1.0, 2.0)
1	135 (53.1%)
≥2	119 (46.9%)
Catheter placement history	
Left internal jugular vein	19 (7.5%)
Right internal jugular vein	245 (96.5%)
Bilateral catheter placement	10 (3.9%)
Current catheter status	
No catheter	36 (14.2%)
With catheter	218 (85.8%)
Catheter type	
Split tip	16 (7.3%)
Step tip	103 (47.2%)
Symmetric tip	83 (38.1%)
Temporary catheter	16 (7.3%)
Catheter tip position	
Upper segment of RA	49 (22.5%)
Middle segment of RA	62 (28.4%)
Lower segment of RA	29 (13.3%)
Other (outside RA)	78 (35.8%)
Main reason for enrollment	
CVC flow reduction	100 (39.4%)
Other non-obstructive reasons	128 (50.4%)
Facial/extremity edema or varicose veins	26 (10.2%)

Note: Percentages of catheter tip positions were based on patients with catheters in situ (*n* = 218). Abbreviation: BMI, body mass index; CVC, central venous catheter; RA, right atrium.

**Table 2:** Comparison of right atrial thrombus detection with TTE and DSA (*n*).

	DSA+	DSA−	Total
TTE+	23	21	44
TTE−	14	196	210
Total	37	217	254

Note: DSA, digital subtraction angiography; TTE, transthoracic echocardiography.

## Data Availability

The raw clinical data generated and analyzed during this study are not publicly available due to patient privacy and ethical restrictions but are available from the corresponding author upon reasonable request.

## Abbreviations

The following abbreviations are used in this manuscript:

AVF	arteriovenous fistula
CRAT	catheter-related right atrial thrombosis
CT	computed tomography
CVC	central venous catheter
DSA	digital subtraction angiography
ICC	intraclass correlation coefficient
IQR	interquartile range
MHD	maintenance hemodialysis
MRI	magnetic resonance imaging
OR	odds ratio
TEE	transesophageal echocardiography
TTE	transthoracic echocardiography
